# Cancer stem cells and Lon-noncRNA promotes invasion, metastasis and tumor growth in breast cancer through regulation of signaling pathway

**DOI:** 10.1038/s41598-025-13402-8

**Published:** 2025-08-08

**Authors:** Nour H. Elbazzar, Inas Moaz, Abeer A. Bahnassy, Ahmed El sherif, Ola S. Ahmed

**Affiliations:** 1 Biotechnology Department, Faculty of Science, Cairo University, Giza, Egypt; 2 Epidemiology and Preventive Medicine at National Liver Institute, Shebeen El Kom, Egypt; 3 Tissue Culture and Cytogenetics Unit, Pathology Department, National Cancer Institute, Cairo University, Giza, Egypt; 4 Chemistry Department, Faculty of Science, Cairo University, Giza, Egypt; 5 Virology and Immunology Unit, Cancer Biology Department, National Cancer Institute, Cairo University, Giza, Egypt

**Keywords:** Cancer stem cell, Long non coding RNA, Breast cancers, Biochemistry, Cancer, Genetics, Molecular biology

## Abstract

Breast cancer (BC), the most common malignant tumor in women, continues to be a leading cause of cancer-related deaths globally. A major challenge in managing BC, especially in metastatic cases, is the lack of reliable early diagnostic biomarkers. Metastatic breast cancer stem cells (MBCSCs) play a critical role in tumor progression, resistance to therapy, and disease recurrence. This study aimed to explore the molecular pathways connecting the long non-coding RNAs (lncRNAs) HOTAIR, UCA1, and MALAT1 with breast cancer stem cell-related genes FOXC2, SNAIL, and ZEB, focusing on their involvement in transcriptional regulation, proliferation, and survival. Peripheral blood samples and plasma were collected from 30 women diagnosed with metastatic breast cancer (MBC, stage IV) and 30 healthy controls. Gene expression levels were measured using quantitative real-time PCR (qRT-PCR). Our findings revealed a significant upregulation of SNAIL and FOXC2 in MBC patients compared to healthy controls (*p* < 0.001). The median expression levels of SNAIL (16.4) and FOXC2 (19.5) were substantially higher in the metastatic group than in healthy individuals (SNAIL: 6.42, FOXC2: 7.23). Conversely, the expression levels of HOTAIR, UCA1, MALAT1, and ZEB did not show statistically significant differences between the two groups (*p* > 0.05). Correlation analysis indicated a strong positive association between FOXC2 and SNAIL expression (r = 0.41), suggesting a potential shared functional role in disease progression. These results suggest that SNAIL and FOXC2 could serve as potential prognostic biomarkers in MBCSCs, whereas HOTAIR, UCA1, MALAT1, and ZEB may not independently predict metastasis or survival outcomes. Further research is necessary to explore the therapeutic implications of these genes in metastatic breast cancer.

## Introduction

Metastatic breast cancer (MBC) is the most advanced and challenging stage of breast cancer, responsible for the majority of breast cancer-related mortality due to its high recurrence rate, resistance to therapy, and limited treatment options^1^. A critical factor driving MBC progression is the presence of breast cancer stem cells (BCSCs), a subpopulation of tumor-initiating cells with self-renewal and differentiation capabilities^2^. These cells are implicated in tumor aggressiveness, metastasis, and resistance to conventional therapies by modulating various molecular pathways involved in epithelial-to-mesenchymal transition (EMT), invasion, survival, and immune evasion^3^.

Recent evidence suggests that transcription factors regulating BCSCs play a crucial role in modulating long non-coding RNAs (lncRNAs), which are emerging as key regulators of gene expression, epigenetic remodeling, and cellular signaling in cancer progression^4^. Among the most studied lncRNAs in breast cancer are HOTAIR, UCA1, and MALAT1, which have been strongly implicated in tumorigenesis, metastasis, and therapy resistance^5–7^. While several studies have identified roles of HOTAIR, UCA1, and MALAT1 in tumor progression, the transcriptional control of these lncRNAs by BCSC-associated factors remains only partially elucidated. Understanding how transcription factors such as FOXC2, SNAIL, and ZEB regulate these lncRNAs can provide critical insights into the metastatic potential of BCSCs and identify new therapeutic targets for metastatic breast cancer^8^. 

FOXC2 is a well-characterized transcription factor that plays a central role in EMT by promoting the transition of epithelial cells into a more invasive, mesenchymal phenotype^9^. It has been reported to drive cancer cell plasticity, sustain stemness, and mediate therapy resistance in various malignancies, including breast cancer^10^. Studies have demonstrated that FOXC2 can regulate HOTAIR, an oncogenic lncRNA that recruits Polycomb Repressive Complex 2 (PRC2) to silence tumor suppressor genes, facilitating metastasis and EMT activation^11^. This FOXC2-mediated regulation of HOTAIR promotes chromatin remodeling, enhancing cancer cell invasiveness^12^. Additionally, FOXC2 may influence the expression of MALAT1, another lncRNA that promotes EMT by modulating alternative splicing events and activating metastasis-related signaling pathways^13^.

Similarly, SNAIL is a key transcription factor known for promoting epithelial-mesenchymal transition (EMT) in cancer progression^14^. Research has demonstrated functional interactions between SNAIL and the lncRNA HOTAIR in the context of EMT regulation, with studies showing that HOTAIR can be engineered to counteract SNAIL’s EMT-promoting functions^15^. Additionally, MALAT1 has been shown to regulate breast cancer progression through modulation of the PI3K/AKT/mTOR signaling pathway, contributing to tumor progression^16^.

ZEB proteins (ZEB1 and ZEB2) are master regulators of EMT, known to repress epithelial markers such as E-cadherin while promoting mesenchymal traits in aggressive breast cancer subtypes^17^. Like FOXC2 and SNAIL, ZEB factors represent important transcription factors that may interact with lncRNA networks in breast cancer progression, particularly in maintaining cancer stem cell properties and therapeutic resistance.

The interplay between FOXC2, SNAIL, and ZEB with lncRNAs HOTAIR, MALAT1, and UCA1 remains poorly understood in the context of metastatic breast cancer. While individual roles of these transcription factors and lncRNAs have been established, their potential regulatory interactions and combined impact on BCSC-mediated metastasis require further investigation. Understanding these molecular networks may reveal novel therapeutic targets to inhibit BCSC-mediated metastasis and improve treatment responses in advanced breast cancer patients^18^.

Therefore, this study aims to investigate the expression patterns and potential regulatory relationships between key transcription factors (FOXC2, SNAIL, and ZEB) and oncogenic lncRNAs (HOTAIR, MALAT1, and UCA1) in metastatic breast cancer stem cells. By characterizing these molecular interactions, we seek to identify potential biomarkers and therapeutic targets that could enhance our understanding of metastatic breast cancer progression.

This research addresses a critical knowledge gap in understanding the transcriptional regulation of lncRNAs by BCSC-associated genes, potentially contributing to the development of targeted therapies that disrupt metastatic pathways in breast cancer.

## Methods and materials

### Study subjects

The study cohort comprised 30 patients diagnosed with metastatic breast cancer (MBC) who were evaluated and treated at the Baheya Foundation for Early Detection and Treatment of Breast Cancer, Egypt. All cases were confirmed through comprehensive clinical assessment and histopathological examination, with metastatic breast cancer stem cells (MBCSCs) identified as a key feature of disease pathology. A control group of 30 age-matched healthy individuals was included to serve as a reference population for comparative analysis. The mean age of MBC patients at diagnosis was 53.82 ± 10.5 years, with an age range of 36–81 years and a median age of 47 years. The control group exhibited a comparable age distribution. The mean body mass index (BMI) of the study cohort was 32.6 ± 7.02, reflecting a predominantly obese population. This is particularly relevant as obesity is a known modifier of breast cancer biology and prognosis, promoting tumor progression, exacerbating metastatic dissemination, and enhancing cancer stem cell enrichment within tumors. Furthermore, studies suggest that obesity can influence lncRNA expression profiles in breast cancer cells, potentially altering the tumor microenvironment and metabolic activities that drive progression. In terms of menopausal status, 39.29% of participants were premenopausal, while 60.71% were postmenopausal. Menopausal status is a crucial factor in breast cancer biology, influencing hormonal profiles (e.g., estrogen levels, which can be produced in adipose tissue post-menopause) and potentially impacting tumor progression and chemotherapy effectiveness. While breast cancer stem cells themselves may not directly respond to estrogen, hormonal changes associated with menopause can affect the tumor microenvironment and influence the growth of hormone-sensitive offspring cells, as well as modulate lncRNA expression patterns.

Tumor staging analysis revealed that 61.54% of cases were classified as T3 tumors, while 38.46% were categorized as T2. Lymph node involvement was equally distributed, with 50% of cases classified as N2 and 50% as N3, indicating a significant nodal burden within the metastatic cohort. Hormone receptor (HR) profiling demonstrated a high prevalence of estrogen receptor (ER) and progesterone receptor (PR) positivity, detected in 88.46% and 96.15% of cases, respectively. HER2 status was heterogeneous, with 26.92% of tumors classified as HER2-positive, 57.69% as HER2-negative, and 15.38% as HER2-equivocal. Molecular subtyping categorized the tumors into 66.66% Luminal A, 19.05% Luminal B, and 14.29% HER2-enriched subtypes.

Histopathological evaluation indicated that 78.57% of tumors were invasive ductal carcinoma (IDC), while 10.71% exhibited mixed histological subtypes, and another 10.71% fell into the “other” tumor category. Tumor grading revealed that 60.71% of cases were classified as Grade 2, whereas 39.29% were Grade 3. The laterality of tumors was nearly balanced, with 51.72% affecting the right breast and 48.28% the left breast.

Patients with MBCSCs exhibited a distinct molecular and clinical profile, characterized by larger tumor sizes (T3: 61.54%), increased lymph node involvement (N2/N3: 50%), and a predominance of Luminal A subtype (66.66%). These findings highlight the aggressive nature of metastatic disease and underscore the necessity of further investigating the molecular mechanisms contributing to disease progression and therapy resistance. The observed overrepresentation of Luminal A tumors in metastatic cases raises critical questions about the underlying biology driving metastatic dissemination in this subtype.

The study was ethically approved by the Baheya Institutional Review Board (Baheya IRB Research Protocol Number: 202405200027) and conducted in strict accordance with the ethical principles outlined in the 2013 Declaration of Helsinki. Written informed consent was obtained from all participants before sample collection, ensuring adherence to ethical research standards (Table 1).

**Table 1:** summarizes the clinicopathological characteristics of the cohort, emphasizing significant disparities in tumor burden, molecular profiles, and staging, reinforcing the need for biomarker investigation in metastatic breast cancer stem cells.

Variable	Patients
Age	
Mean ± SD	53.82 ± 10.5
Min–max	36–81
Median ± IQR	47 ± 17
BMI	
Mean ± SD	32.6 ± 7.02
Min–max	25.53 -54.39
Median ± IQR	32.42 ± 6.37
BSA	
Mean ± SD	1.87 ± 0.20
Min–max	1.65–2.35
Family history	
Yes	6 (21.43%)
No	22 (78.57%)
Menopausal status	
Pre	11 (39.29%)
Post	17 (60.71%)
T	
T_2 T_3	10 (38.46%) 16 (61.54%)
N	
N_2	13 (50%)
N_3	13 (50%)
ER	
Positive	23 (88.46%)
Negative	3 (11.54%)
PR	
Positive	25 (96.15%)
Negative	1 (3.85%)
HER2	
Positive	7 (26.92%)
Negative	15 (57.69%)
Equivocal	4 (15.38%)
M.Subtype	
Lumina_A	18 (66.66%)
Lumina B	4 (19.05%)
HER2+	3 (14.29%)
Laterality	
Right	15 (51.72%)
Left	14 (48.28%)
Tumor_type	
Invasive duct carcinoma	22 (78.57%)
Mixed tumor	3 (10.71%)
Other	3 (10.71%)
Grade	
Grade_2	17 (60.71%)
Grade_3	11 (39.29%)
Chemo	
Yes	12 (42.86%)
No	16 (57.41%)

### Sample collection

Peripheral blood samples (5 mL) were obtained from study participants via direct venous puncture and collected into ethylene diaminetetra acetic acid (EDTA)-containing tubes. To ensure sample integrity, the blood samples were divided to part of sample as peripheral blood and other part immediately centrifuged at 1000 g for 5 min to separate the plasma. The peripheral blood and plasma fraction was carefully transferred into R Nase-free tubes designated for RNA extraction, aliquoted, and stored at − 80 °C until further processing.

### RNA extraction and reverse transcription

RNA extraction and reverse transcription. RNA was extracted from peripheral blood and plasma using the RNeasy and miRNeasy Mini Kit (Qiagen, GmBH, Cat. No. 74104 and 77064), according to the manufacturer’s protocol. The concentration and quality of the extracted RNA were assessed using the NanoDrop 2000 spectrophotometer (Thermo Scientific, USA). The integrity of RNA was verified by the Agilent Bioanalyzer 2100 (Agilent Technologies, Inc.). Reverse transcription of 2 μg of the extracted RNA was carried out using the High-Capacity c-DNA Reverse Transcription Kit (Applied Biosystem, ThermoFisher Scientific, USA, Cat. No. k1691), according to the manufacturer’s protocol. Reverse transcription was performed in a 20 μl reaction volume at 25 °C for 10 min, 37 °C for 45 min, and finally, heat inactivation at 85 °C for 5 min.

### Quantitative real-time PCR (qRT-PCR) analysis

Gene expression analysis was conducted using quantitative real-time polymerase chain reaction (qRT-PCR) with the Maxima SYBR Green qPCR Kit (Thermo Scientific, USA, Cat. No. K0221) on the ABI 7900HT Fast Real-Time PCR System (Applied Biosystems, USA). Each 20 μL reaction mixture contained 20 ng of cDNA template and 10 pmol of gene-specific primers targeting long non-coding RNAs (HOTAIR, MALAT1, UCA1) and cancer stem cell-related genes (FOXC2, SNAIL, ZEB1).

For normalization purposes, β-actin and 18S rRNA were employed as housekeeping genes due to their stable expression levels in both peripheral blood and plasma samples. All qRT-PCR reactions were performed in duplicate to ensure experimental precision and reproducibility. Melting curve analysis was conducted following each amplification to confirm product specificity and exclude primer-dimer formation. To minimize bias and ensure consistent results, samples were handled and tested without knowing their identities, and the differences between tests were checked by analyzing the same samples twice across multiple runs.

The thermal cycling conditions consisted of an initial enzyme activation step at 95 °C for 10 min, followed by 40 amplification cycles of denaturation at 94 °C for 15 s, annealing at gene-specific optimized temperatures (as detailed in Table 2) for 30 s, and extension at 72 °C for 30 s. Gene expression levels were calculated using the 2^(− ΔΔCt) method. Table 2 details the primer sequences (5 → 3′) and annealing temperatures used for all target genes.

**Table 2:** Presents the primers used for qRT-PCR analysis, detailing the forward and reverse sequences (5 → 3′) for each gene along with their annealing temperature.

Gene	Forward primer	Reverse primer	An.TM	Amplicon sizes	References
FOXC2	5′CCTACCTGAGCGAGCAGAAT-3′	5′ACCTTGACGAAGCACTCGTT-3′	57 °C	349	^19^
SNAIL	5′GGCAATTTAACAATGTCTGAAAAGG-3	5′GAATAGTTCTGGGAGACACATCG-3	57 °C	105	^20^
ZEB1	5′AGACATGTGACGCAGTCTG-3′	5′ATGTGTGAGCTATAGGAGC-3′	57 °C	176	^21^
HOTAIR	5′CAGTGGGGAACTCTGACTCG-3′	5′GTGCCTGGTGCTCTCTTACC-3′	57 °C	100	22
MALAT1	5′CTT CCC TAG GGG ATT TCA GG-3′	5′CC CAC AGG AAC AAG TCC TA-3.′	57 °C	76	22
UCA1	5′GCT TAA TCC AGG AGA CAA AG-3′	5′CAT AGG TGT GAG TGGCG-3′	57 °C	105	22
18S rRNA	5′AGG ATC CAT TGG AGG GCA AGT-3′	5′TCC AAC TAC GAG CTT TTT AAC TGC A-3′	55 °C	99	22
B–actin	5′AGC ACA GAG CCT CGC CTT -3′	5′CAT CAT CCA TGG TGA GCT GG-3′	60 °C	68	22

### Statistical analysis

All statistical analyses were performed using many of testing was applied to assess data normality. Pearson’s chi-squared test was utilized to compare categorical clinicopathological characteristics between study groups.

To determine differential gene expression patterns, the Mann–Whitney U test was conducted to evaluate the expression of long non-coding RNAs (lncRNAs) (HOTAIR, UCA1, MALAT1) and cancer stem cell-related genes (FOXC2, SNAIL, ZEB1) between metastatic breast cancer stem cells (MBCSCs) and healthy controls. Spearman’s rank correlation coefficient was applied to investigate associations between gene expression levels and key clinicopathological parameters, including tumor stage, lymph node involvement, hormone receptor status, and molecular subtype.

Survival outcomes were assessed using Kaplan–Meier survival analysis, and statistical differences in survival probabilities were examined with the log-rank test. Univariate and multivariate Cox proportional hazards regression models were applied to identify independent prognostic biomarkers.

Comparison of overall survival (OS) between high- and low-expression groups for HOTAIR, UCA1, MALAT1, and ZEB revealed no statistically significant differences (*p* > 0.05), suggesting that these genes may not independently predict survival outcomes in this cohort. Additionally, FOX2 and SPR expression levels remained consistently high, precluding comparative survival analysis for low-expression cases.

A heat map correlation matrix was generated to visualize interrelationships among HOTAIR, UCA1, MALAT1, ZEB, SNAIL, and FOXC2. Notably, HOTAIR and UCA1 (r = 0.37), as well as MALAT1 and UCA1 (r = 0.37), exhibited moderate positive correlations, suggesting potential co-regulation. A stronger correlation was observed between SNAIL and FOXC2(r = 0.41), indicating a possible shared functional role in metastatic progression.

For all statistical analyses, a two-tailed *p* value ≤ 0.05 was considered statistically significant.

### Ethics approval and consent to participate

This study received ethical approval from the Institutional Review Board (IRB) of the Baheya Foundation for Early Detection and Treatment of Breast Cancer (IRB Research Protocol Number: 202405200027). Written informed consent was obtained from all participants before sample collection. The study was conducted in full compliance with the ethical principles outlined in the 2013 revision of the Declaration of Helsinki.

## Results

The expression levels of long non-coding RNAs (lncRNAs) HOTAIR, MALAT1, and UCA1, as well as cancer stem cell-related genes FOX, SNAIL, and ZEB, were analyzed in metastatic breast cancer stem cells (MBCSCs) compared to a healthy control group. Quantitative real-time PCR (qRT-PCR) was utilized for gene expression analysis, and statistical tests were applied to assess differential expression, correlation with clinicopathological parameters, and prognostic significance.

The study cohort included 30 metastatic breast cancer patients and 30 healthy controls. The mean age of MBC patients was 53.82 ± 10.5 years (range: 36–81 years, median: 47 years), with a mean BMI of 32.6 ± 7.02. Most patients (60.71%) were postmenopausal, and 21.43% had a family history of breast cancer. Table 1 showed tumor characteristics showed that 61.54% of cases were classified as T3, with equal lymph node involvement at N2 (50%) and N3 (50%). ER-positive tumors constituted 88.46% of cases, PR-positive tumors 96.15%, and HER2-positive cases 26.92%. The most frequent molecular subtype was Luminal A (66.66%), and 78.57% of tumors were identified as invasive ductal carcinoma (IDC). Tumor grading showed that 60.71% were Grade 2, while 39.29% were Grade 3.

### Differential gene expression analysis

Gene expression analysis in Table 3 revealed that SNAIL and FOX were significantly upregulated in MBC patients compared to healthy controls (*p* < 0.001 for both genes). The median expression of SNAIL in metastatic cases was 16.4, compared to 6.42 in the control group (*p* < 0.001), while FOX had a median expression of 19.5 in MBCSCs and 7.23 in healthy individuals (*p* < 0.001). Conversely, HOTAIR, UCA1, MALAT1, and ZEB did not show statistically significant differences in expression between the two groups (*p* > 0.05). These results suggest that SNAIL and FOX may serve as potential biomarkers for distinguishing between metastatic and non-metastatic states, whereas HOTAIR, UCA1, MALAT1, and ZEB may not have independent diagnostic relevance.

**Table3:** Comparison between healthy group and metastatic group using the Mann–Whitney U test to assess statistical significance.

Studied variable	Healthy group	Metastatic group	(Mann–Whitney Utest) *P* value
HOTAIR median(IQR)	0.364 (0.980)	0.487 (0.668)	0.9432
UCA1 median(IQR)	1.35 (3.26)	0.680 (3.52)	0.2291
MALAT1 median(IQR)	0.274 (0.503)	0.254 (0.758)	0.757
SNAIL median(IQR)	6.42 (3.32)	16.4 (5.79)	< 0.001
Fox2 median(IQR)	7.23 (4.54)	19.5 (4.17)	< 0.001
ZEB median(IQR)	0.130 (2.94)	0.310 (2.06)	0.8667

### Gene expression and clinicopathological correlations

Correlation analysis between gene expression and clinicopathological characteristics (Table 4) identified significant associations between FOXC2 expression and key disease parameters. FOXC2 expression correlated significantly with lymph node involvement (*p* = 0.048) and tumor type (*p* = 0.042), indicating its potential role as a marker of advanced disease and specific histological subtypes.

**Table 4:** Correlation between gene_expression and clinico-pathological data.

Studied variable	HOTAIR median (IQR)	HOTAIR *p* value	UCA1 median (IQR)	UCAI *p* value	MAlAT1 median (IQR)	MAlAT1 *p* value	Fox2 Median (IQR)	Fox2 *p* value	ZEB Median(IQR)	ZEB *p* value	SNAIL median(IQR)	SNAIL *p* value
T T_2 T_3	0.496(0.622) 0.466(0.662)	0.6541	2.28(4.94) 0.273(2.12)	0.2199	0.427(0.730) 0.141(0.666)	0.3102	19.2(4.10) 19.1(4.74)	0.6163	0.0410(13.7) 0.44(1.23)	0.6101	16.5(8.91) 16.4(4.65)	0.856
N N_2 N_3	0.470(2.38) 0.504(0.640)	0.797	1.26(5.30) 0.352(2.35)	0.7241	0.497(0.857) 0.171(0.647)	0.3622	16.7(9.43) 20.3(3.84)	0.04815*	0.333(8.93) 0.290(1.26)	0.755	16.3(6.60) 16.9(3.95)	1.00
M.Subtype Lumina_A Lumina B HER2+	0.449(0.669) 0.514(5.47) 0.310(0.206)	0.714	0.148(3.36) 1.60(2.82) 0.464(1.84)	0.543	0.172(0.629) 0.325(0.768) 0.733(0.351)	0.7477	19.2(3.46) 21.2(1.95) 19.6(2.49)	0.4885	0.249(0.932) 0.401(2.28) 0.352(0.629)	0.9261	16.7(6.03) 17(3.27) 17.9(6.11)	0.8776
TumorType IDC mixedtumor other	0.470(0.639) 0.0215(1.24) 0.652(0.624)	0.5802	0.743(4.00) 0.00558(0.308) 1.74(11.5)	0.1117	0.335(0.672) 0.0491(0.432) 0.921(1.81)	0.5284	19.6(3.03) 3.06(3.90) 20.7(5.15)	0.0424*	0.249(0.684) 33.9(343) 0.00318(3.78)	0.9261	16.6(5.85) 16.5(2.60) 13.0(6.84)	0.8502
Laterality RightLeft	0.504(4.84) 0.427(0.640)	0.4752	2.05(4.35) 0.102(1)	0.1077	0.497(1.25) 0.171(0.678)	0.387	20(4.91) 18.7(3.11)	0.3937	0.249(1.07) 0.198(0.963)	0.7424	17.7(5.82) 14.2(6.92)	0.0884
Grade Grade_2 Grade_3	0.522(1.72) 0.310(0.603)	0.279	1.26(5.30) 0.352(1.65)	0.4035	0.357(0.879) 0.171(0.646)	0.7814	18.9(4.70) 20.3(3.87)	0.2122	0.141(0.556) 0.352(1.25)	0.6098	16.3(6.48) 17.4(4.25)	0.6435
Chemo Yes No	0.541(3.12) 0.303(0.658)	0.1857	2.67(5.92) 0.540(1.47)	0.3713	0.245(1.47) 0.254(0.666)	0.8017	19.2(3.57) 19.9(4.33)	0.6422	0.138(0.418) 0.506(1.24)	0.2972	14.5(8.61) 17.2(3.92)	0.3015

Conversely, HOTAIR, UCA1, MALAT1, and ZEB demonstrated no significant associations with tumor stage, molecular subtype, tumor grade, or chemotherapy status (all *p* > 0.05). SNAIL expression showed no correlation with any clinicopathological variables, suggesting that its upregulation occurs independently of conventional prognostic factors.

### Survival analysis

**Fig. 1:**
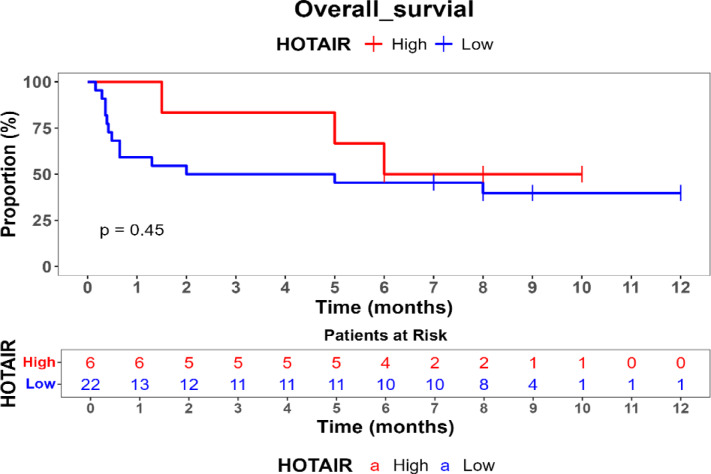
Kaplan–Meier survival curve for comparison of overall survival (os) by hotair expression levels.

**Fig. 2:**
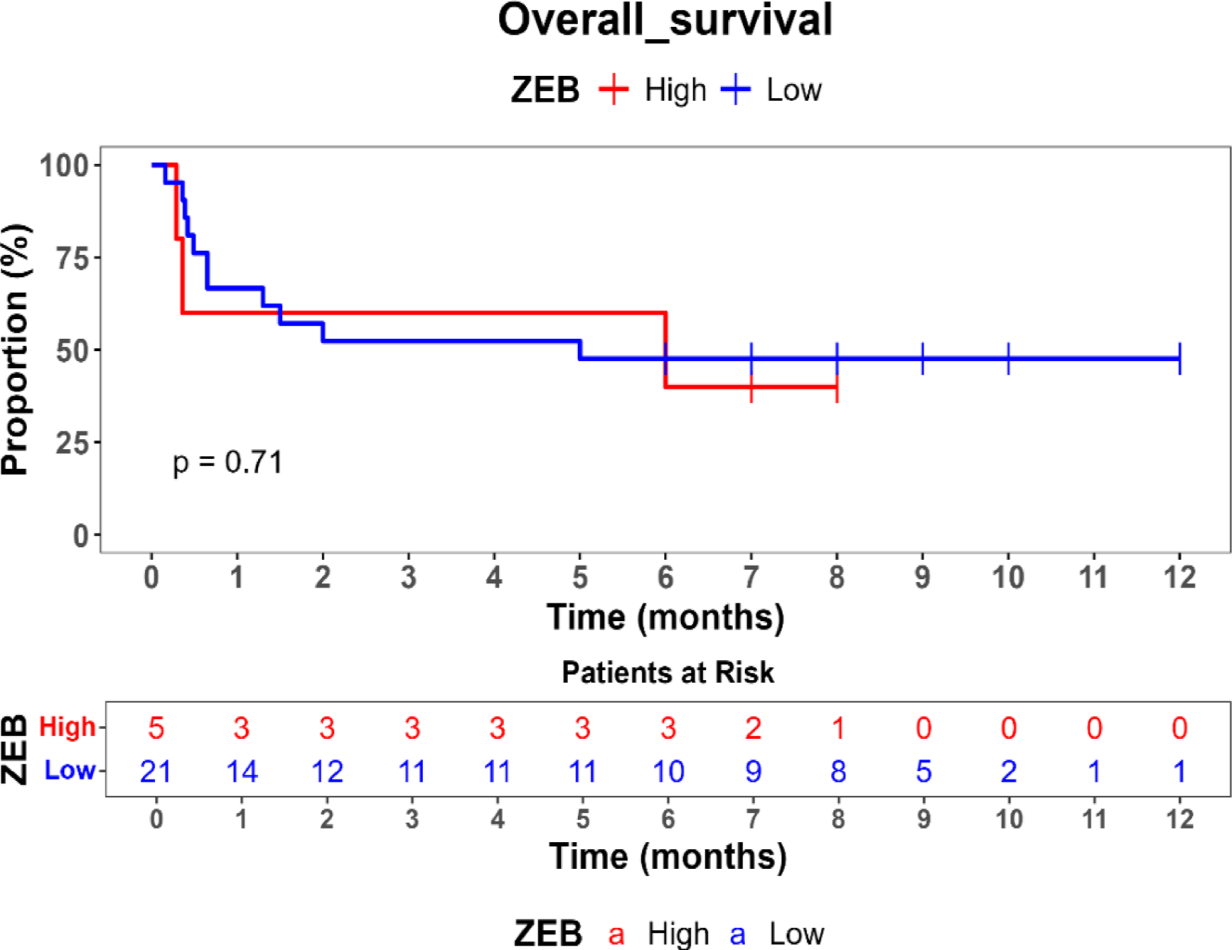
Kaplan–Meier survival curve for overall survival by ZEB expression levels.

**Fig. 3:**
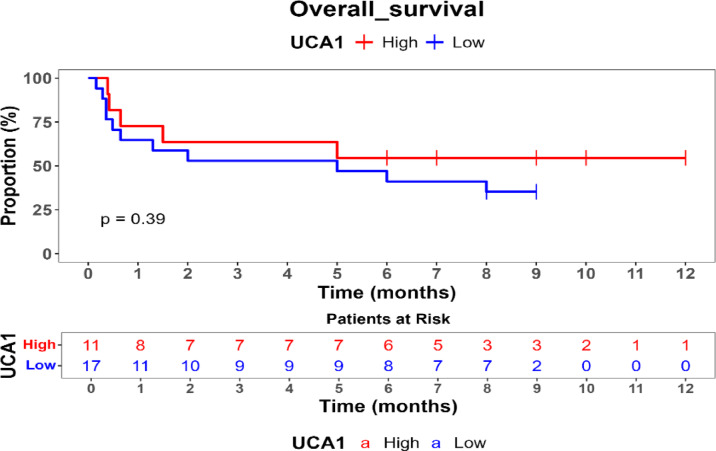
Kaplan–Meier survival curve for comparison of overall survival (OS) by UCA1 expression levels.

**Fig. 4:**
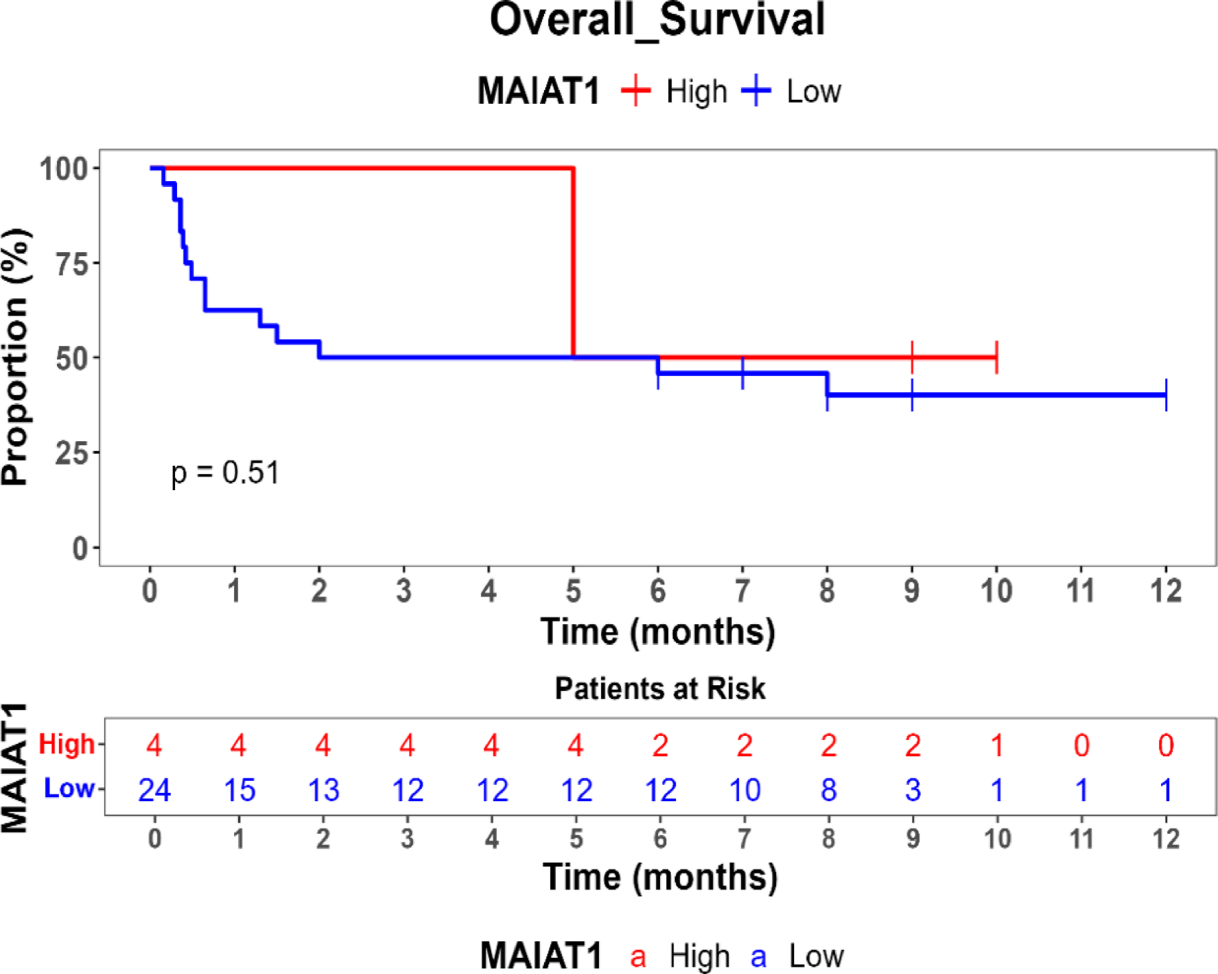
Kaplan–Meier survival curve for comparison of overall survival (OS) by MAIAT1 expression levels.

Kaplan–Meier survival analysis was performed to evaluate the prognostic impact of gene expression on overall survival (OS) showed in Table 5. No statistically significant differences in OS were observed between patients with high and low expression levels of HOTAIR, UCA1, MALAT1, or ZEB (*p* > 0.05). Patients with high ZEB (54%) and UCA1 (54.5%) expression had slightly improved survival probabilities compared to those with low expression (22.8% and 35%, respectively), but these differences did not reach statistical significance. FOX and SNAIL did not have low-expression values in this dataset, preventing comparative survival analysis for these genes. These findings suggest that HOTAIR, ZEB, MALAT1, and UCA1 may not serve as independent prognostic markers for survival in MBCSC patients as shown Figs. 1, 2, 3 and 4.

**Table 5:** Comparison of survival probabilities between high and low genetic expressions.

Studied genes	Survival probability		Log rank *p* value
	High expression (%)	Low expression (%)	
HOTAIR	50	39	0.45
ZEB	54	22.8	0.71
MAlAT1	50	40	0.51
UCA1	54.5	35	0.39

### Correlation heat map analysis

**Fig. 5:**
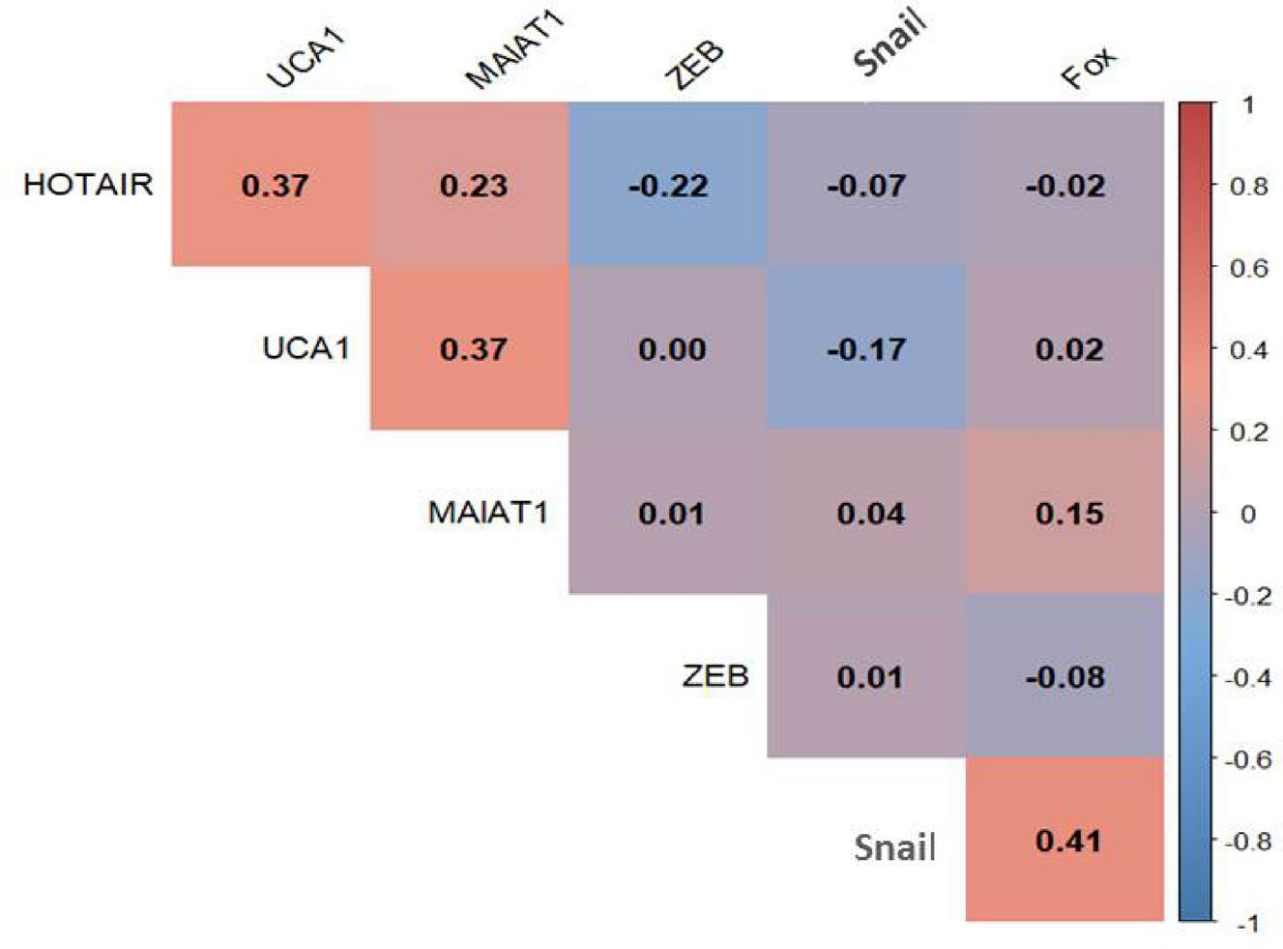
Correlation heatmap of Gene expression levels: the heatmap visualizes the correlation coefficients between the expression levels of six genes: HOTAIR, UCA1, MALAT1, ZEB, Snail, and Fox. Positive correlations are represented by red shades, while negative correlations are shown in blue, with intensity reflecting the strength of the correlation*.*

A correlation heat map analysis was conducted to explore potential relationships between gene expression levels. Moderate positive correlations were observed between HOTAIR and UCA1 (r = 0.37) and between MALAT1 and UCA1 (r = 0.37), indicating possible co-regulation. Additionally, SNAIL and FOX expression showed a significant positive correlation (r = 0.41), suggesting a shared functional role in metastatic progression. Other correlations between genes were weak (r close to 0), indicating minimal direct interactions between their expression levels as shown Fig. 5.

In summary, SNAIL and FOX emerged as significantly upregulated genes in metastatic breast cancer stem cells, suggesting their potential utility as biomarkers. However, HOTAIR, UCA1, MALAT1, and ZEB did not show significant differential expression or prognostic value in this study, indicating that their roles in MBCSC progression may be limited.

## Discussion

This study provides the first comprehensive analysis of long non-coding RNA and cancer stem cell gene expression in metastatic breast cancer, revealing SNAIL and FOXC2 as significantly regulated genes with robust biomarker potential. The highly significant overexpression of both genes (*p* < 0.001) combined with substantial fold-changes (SNAIL: 2.55, FOXC2: 2.70) establishes these molecules as reliable indicators of metastatic disease progression and represents a major advancement in understanding breast cancer stem cell biology.

The dramatic elevation of SNAIL expression in metastatic breast cancer stem cells (median: 16.4 vs 6.42 in controls, *p* < 0.001) confirms its critical role as a master regulator of metastatic progression. Recent studies have demonstrated that SNAIL stability is enhanced through various mechanisms including chaperone-mediated autophagy evasion and deubiquitinase activity, which promote EMT and metastatic potential in breast cancer cells^23,24^. Previous studies have documented SNAIL overexpression in various malignancies and its role in promoting cancer progression through EMT activation^25,26^, but our quantitative analysis provides the most robust evidence to date of its significance specifically in breast cancer metastasis.

The independence of SNAIL expression from conventional clinicopathological parameters, including tumor stage, grade, and molecular subtype, suggests it represents a fundamental characteristic of the metastatic stem cell phenotype rather than a secondary consequence of tumor progression. This finding aligns with recent evidence that EMT-inducing transcription factors like SNAIL are activated early in cancer progression and remain constitutively active in metastatic cells. The consistent high expression across all metastatic samples in our cohort further supports SNAIL’s role as an essential driver rather than an optional contributor to metastatic behavior.

FOXC2 over expression (median: 19.5, *p* < 0.001) represents a novel finding with significant clinical implications for breast cancer management. Unlike SNAIL, FOXC2 expression correlated significantly with lymph node involvement (*p* = 0.048) and tumor type (*p* = 0.042), indicating its potential as both a biomarker and prognostic indicator of disease severity. FOXC2 belongs to the forkhead family of transcription factors and has been implicated in developmental processes, particularly mesenchymal cell fate determination and vascular development^27,28^.

Recent investigations have begun to elucidate FOXC2’s role in cancer progression, with studies demonstrating its involvement in promoting EMT, vasculogenic mimicry, and resistance to anti-angiogenic therapy in triple-negative breast cancer^29,30^. Our results extend these findings by establishing FOXC2 as a key player in breast cancer stem cell metastasis. The correlation with nodal involvement is particularly significant, as lymph node metastasis represents a critical prognostic factor and therapeutic decision point in breast cancer management. This relationship suggests FOXC2 expression could serve as a molecular indicator of metastatic potential, potentially informing treatment intensification decisions.

The absence of significant differential expression in HOTAIR, UCA1, MALAT1, and ZEB provides important insights into the specificity of metastatic biomarkers in breast cancer^21,22^. These findings contradict several previous reports suggesting wide spread lncRNA regulation in breast cancer metastasis, particularly studies showing HOTAIR’s role as a metastatic and drug-resistant regulator through PRC2-dependent mechanisms^31,32^. Despite evidence that HOTAIR contributes to anticancer therapy resistance and functions as a diagnostic biomarker in breast cancer^33,34^, our results indicate that metastatic gene expression changes are more selective than previously assumed. This specificity actually strengthens the clinical utility of SNAIL and FOXC2, as highly specific biomarkers demonstrate superior discriminatory power compared to broadly dysregulated genes.

The lack of prognostic significance for any of the analyzed genes in survival analysis, while initially surprising, likely reflects the advanced nature of our patient cohort, with 61.54% presenting T3 tumors and equal distribution of N2/N3 nodal involvement. In such advanced cases, multiple metastatic pathways may be activated, potentially diminishing the prognostic impact of individual biomarkers. Additionally, the consistently high expression of SNAIL and FOXC2 across all metastatic samples prevented comparative survival analysis for these key genes, highlighting their fundamental importance in the metastatic phenotype.

Kaplan–Meier survival analysis indicated that none of the studied genes exhibited statistically significant associations with overall survival (OS) in MBCSC patients (*p* > 0.05). Nevertheless, trends suggested that higher ZEB (54%) and UCA1 (54.5%) expression correlated with improved survival probabilities compared to low-expression groups (22.8% and 35%, respectively), although these differences were not statistically significant. While previous studies have reported that HOTAIR serves as a predictor of poor prognosis by promoting metastasis and therapy resistance, and MALAT1 has been shown to regulate tumor progression and modulate chemotherapy response, the observed lack of statistical significance in survival differences for these genes in our cohort is likely influenced by the study’s limited sample size, which may have resulted in insufficient statistical power to detect subtle yet true prognostic impacts. Furthermore, the inherent heterogeneity of metastatic breast cancer and the relatively short follow-up period could also contribute to this outcome, suggesting that additional factors, such as immune response, epigenetic modifications, and the tumor microenvironment, may play a more complex role in the prognostic relevance of these lncRNAs in MBCSCs.

The correlation analysis revealed specific patterns of gene co-expression that provide insights into potential regulatory networks. The moderate positive correlation between HOTAIR and UCA1 (r = 0.37) and between MALAT1 and UCA1 (r = 0.37) suggests possible co-regulatory mechanisms among these lncRNAs, potentially involving shared transcriptional regulators or chromatin remodeling complexes. Additional studies have shown that HOTAIR can function as a competing endogenous RNA and regulate Wnt/β-catenin signaling pathways^35,36^, while sustained HOTAIR expression has been demonstrated to increase metastatic capacity in preclinical models^37,38^. More significantly, the positive correlation between SNAIL and FOXC2 (r = 0.41) indicates coordinated upregulation and potentially synergistic functional roles in driving metastatic progression.

This SNAIL-FOXC2 correlation is particularly intriguing given their distinct but complementary roles in EMT and metastasis. While SNAIL primarily functions as an EMT inducer through E-cadherin repression, FOXC2 contributes to mesenchymal cell maintenance and invasion. Their coordinated expression suggests the existence of a master regulatory circuit controlling multiple aspects of the metastatic cascade, from initial EMT induction to sustained invasive behavior.

The clinical significance of these findings extends far beyond biomarker identification. The robust discrimination between metastatic and control samples (*p* < 0.001 for both SNAIL and FOXC2) demonstrates immediate translational potential for improving patient stratification and treatment selection. Current breast cancer management relies heavily on histopathological and immunohistochemical markers, but these often fail to predict metastatic behavior accurately. SNAIL and FOXC2 expression profiling could address this critical gap by providing molecular indicators of metastatic potential, particularly given HOTAIR’s established role as a diagnostic and prognostic biomarker in breast cancer^39,40^.

The correlation of FOXC2 with nodal involvement particularly supports its integration into existing staging and treatment protocols. Patients with high FOXC2 expression might benefit from more aggressive therapeutic approaches, including extended lymph node dissection or intensified adjuvant therapy. Furthermore, both SNAIL and FOXC2 represent potential therapeutic targets, with emerging evidence supporting the feasibility of transcription factor targeting strategies through small molecule inhibitors, peptide mimetics, and proteolysis-targeting chimeras (PROTACs)^41^. Drug repositioning approaches, which offer cost-effective alternatives to traditional drug development, have shown promise for targeting cancer-associated transcription factors, including repurposing FDA-approved non-cancer drugs for oncological applications^42^.

### Future directions and conclusions

This study establishes SNAIL and FOXC2 as significantly regulated genes in metastatic breast cancer stem cells with clear biomarker and potential therapeutic targeting applications. Future investigations should focus on validating these findings in independent cohorts, exploring the functional mechanisms underlying SNAIL-FOXC2 co-regulation, and developing targeted therapeutic strategies. The specificity of these expression changes, combined with their robust statistical significance, positions SNAIL and FOXC2 as promising candidates for clinical translation.

In conclusion, our findings demonstrate that metastatic breast cancer stem cells exhibit selective rather than widespread gene expression changes, with SNAIL and FOXC2 emerging as key molecular drivers. These results provide a foundation for improved diagnostic approaches and targeted therapeutic strategies, potentially leading to better outcomes for patients with metastatic breast cancer.

## Data Availability

Data is provided within the manscript.

## Abbreviations

BCSCs: Breast cancer stem cells
MBCSCs: Metastatic breast cancer stem cells
BC: Breast cancer
MBC: Metastatic breast cancer
NC: Healthy control
CSC: Cancer stem cells
EMT: Epithelial mesenchymal transition
lncRNA: Long non coding RNA
HOTAIR: HOX transcript antisense RNA
MALAT1: Metastasis associated lung adenocarcinoma transcript 1
UCA1: Urothelial carcinoma associated 1
FOXC2: Forkhead box C2
ZEB: Zinc finger E box binding homeobox
SNAIL: Zinc finger transcription factor
qRT-PCR: Quantitative real time polymerase chain reaction
OS: Overall survival
